# Shape of a recoiling liquid filament

**DOI:** 10.1038/s41598-019-51824-3

**Published:** 2019-10-29

**Authors:** Francesco Paolo Contò, Juan F. Marín, Arnaud Antkowiak, J. Rafael Castrejón-Pita, Leonardo Gordillo

**Affiliations:** 1 School of Engineering and Materials Science, Queen Mary University of London, London, E1 4NS United Kingdom; 2 Departamento de Física, Universidad de Santiago de Chile, Av. Ecuador, 3493 Estación Central, Santiago Chile; 3 Sorbonne Université, CNRS, Institut Jean le Rond ∂’Alembert, F-75005 Paris, France; 4 Saint-Gobain, CNRS, Surface du Verre et Interfaces, F-93303 Aubervilliers, France

**Keywords:** Fluid dynamics, Nonlinear phenomena

## Abstract

We study the capillary retraction of a Newtonian semi-infinite liquid filament through analytical methods. We derive a long-time asymptotic-state expansion for the filament profile using a one-dimensional free-surface slender cylindrical flow model based on the three-dimensional axisymmetric Navier-Stokes equations. The analysis identifies three distinct length and time scale regions in the retraction domain: a steady filament section, a growing spherical blob, and an intermediate *matching* zone. We show that liquid filaments naturally develop travelling capillary waves along their surface and a neck behind the blob. We analytically prove that the wavelength of the capillary waves is approximately 3.63 times the filament’s radius at the inviscid limit. Additionally, the waves’ asymptotic wavelength, decay length, and the minimum neck size are analysed in terms of the Ohnesorge number. Finally, our findings are compared with previous results from the literature and numerical simulations in Basilisk obtaining a good agreement. This analysis provides a full picture of the recoiling process going beyond the classic result of the velocity of retraction found by Taylor and Culick.

## Introduction

**Figure 1:**
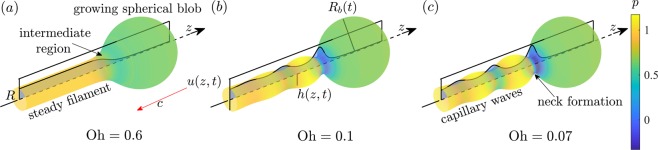
Recoiling of filaments at *t* = 33.8 for (**a**) Oh = 0.6, (**b**) Oh = 0.1, and (**c**) Oh = 0.07. The time *t* is scaled by the capillary time ${t}_{c}=\sqrt{\rho {R}^{3}/\sigma }$. Asymptotic solutions were obtained through a lubrication model. Velocity profiles (black solid lines) and pressure *p* (colour scheme) are also displayed.

The capillary retraction of liquid filaments in a passive ambient fluid is extremely important in several industrial applications, such as atomisation, spraying, inkjet printing, and microfluidics^1^. Additionally, their dynamics are a fundamental problem in nature. A liquid filament, free in the air, retracts by the action of surface tension. This is a classical problem that has been widely studied in the literature through theoretical, numerical and experimental methods^2–10^. However, the first significant studies on this topic were performed by Taylor in 1959^11^ and Culick in 1960^12^, who focused on the capillary retraction of a thin planar fluid sheet. Following basic physics principles, i.e. mass and momentum conservation, they found that the fluid free edge accumulates mass from the film as it retracts, and a growing rim with a circular section is formed. The rim recedes at a retraction speed that tends to a constant value, i.e. the *Taylor-Culick speed*, and is independent of the fluid viscosity as only inertial and capillary effects are considered. In the case of a cylindrical filament of radius *R*, the liquid is collected at the receding tip thus forming a growing spherical blob as depicted in Fig. 1. Mass and momentum balance applied to a domain enclosing the blob yields the Taylor-Culick speed,

1 $$c=\sqrt{\frac{\sigma }{\rho R}},$$

where *σ* is the surface tension and *ρ* is the liquid density. The result is the same as the one for a semi-infinite sheet^11,12^, $c=\sqrt{\sigma /e\rho }$, wher*e e* is the semi-thickness of the sheet. Equation (1) has been discussed elsewhere, e.g., by Hoepffner and Paré in 2013^8^. Other studies by Savva & Bush^13^ showed that the retraction velocity does converge to the Taylor-Culick value after an initial unsteady state, confirming its validity as an asymptotic limit. Indeed, such a powerful prediction is derived from very simple arguments: mass and momentum balance applied at a global scale (net force acting on the growing blob). In addition, Savva & Bush in 2009^13^ and Brenner & Gueyffier in 1999^14^ showed that the liquid viscosity, *μ*, determines the transient-state characteristic time, the interface stability, and the filament profile. The problem is governed by a single dimensionless parameter, the Ohnesorge number ${\rm{Oh}}\equiv \mu /\sqrt{\rho \sigma R}$, which represents the ratio of viscous and capillary forces. Examples of the behaviour of the filament retraction for different Oh are shown in Fig. 1.

In this report, we study the filament interface evolution in the long-time limit, i.e. when the blob radius is much larger than the filament radius, using a local asymptotic analysis. In Section §2, we present a set of two one-dimensional equations for a free surface slender liquid filament under the lubrication approximation. We use this model to analyse the filament dynamics in its quasi-steady state. We show that the long-time asymptotic solution is separated into three regions that have different length and time scales: the steady cylinder far from the blob, the receding blob, and an intermediate matching zone (see Fig. 1). In Section §3, the three different solutions are “stitched” together to provide a unique asymptotic matching solution of the evolving filament shape. Travelling capillary ripples and the neck connecting the blob and the filament, which can be seen under some conditions, are analysed in terms of the Ohnesorge number. These results are important as they provide a full and formal description of the Taylor-Culick regime.

## Filament Equations and Local Solutions

Here, we consider an incompressible axisymmetric Newtonian liquid filament of viscosity *μ* and density *ρ* surrounded by an inert ambient gas with negligible density. The boundaries of the liquid are taken as free surfaces with surface tension *σ*. The filament is a semi-infinite cylinder of radius *R* aligned to the *z*-axis and its free rim recoils as time *t* evolves.

Following the approach of Eggers & Dupont^15^, the system dynamics can be described through a lubrication model. In this long-wavelength limit, the problem is reduced to a set of two coupled equations along the axial direction *z* for two scalar fields: the filament local radius *h*(*z*, *t*) and the mean axial velocity *u*(*z*, *t*). The two equations correspond to local mass conservation and local momentum balance. In dimensionless form (*h*, *z* ~ *R*, *u* ~ *c* and *t* ~ *R*/*c*), these are:

2 $${\partial }_{t}({h}^{2})+{\partial }_{z}({h}^{2}u)=0,$$

3 $${\partial }_{t}({h}^{2}u)+{\partial }_{z}({h}^{2}{u}^{2})={\partial }_{z}(3{\rm{Oh}}\,{h}^{2}{\partial }_{z}u+h[\frac{1+{({\partial }_{z}h)}^{2}+h{\partial }_{zz}h}{{\sqrt{1+{({\partial }_{z}h)}^{2}}}^{3}}])\mathrm{.}$$

Gravity has been neglected and the full non-linear expression for the capillary term has been included as suggested by Eggers^16^.

Under the action of the surface tension, the filament contracts at a rate that asymptotically reaches the Taylor-Culick velocity (*c* = −1 in dimensionless form). For convenience, a reference frame moving at the Taylor-Culick velocity is chosen. The boundary condition for Eqs. (2) and (3) at *z* = −∞ is hence *h* = 1 and *u* = 1, representing the unperturbed filament radius far away from the tip, and the influx velocity at the blob. Likewise, at the tip *z* = *z*_*T*_(*t*), the boundary condition is *h* = 0, ∂_*z*_*h* = −∞ and *u* = ∂_*t*_*z*_*T*_, which sets the symmetry and the kinematic condition at the receding tip. In the next sections, we derive a long-time asymptotic expression for the filament profile from Eqs. (2) and (3).

### Steady filament

Here, we seek solutions for the steady region far from the blob. The following expressions for the filament dimensionless radius and the axial velocity are proposed:

4 $${h}_{f}(z,t)={h}_{f}^{(0)}(z)+{h}_{f}^{(1)}(z,t)+\ldots ,$$

5 $${u}_{f}(z,t)={u}_{f}^{(0)}(z)+{u}_{f}^{(1)}(z,t)+\ldots ,$$

where *h*_*f*_^(1)^(*z*, *t*) and *u*_*f*_^(1)^(*z*, *t*) are higher-order corrections, such that *h*_*f*_^(1)^/*h*_*f*_^(0)^, *h*_*f*_^(2)^/*h*_*f*_^(0)^, … etc, and *u*_*f*_^(1)^/*u*_*f*_^(0)^, *u*_*f*_^(2)^/*u*_*f*_^(0)^, … etc, vanish as *t* → ∞. By replacing these expressions into Eqs. (2) and (3), we obtain, at the leading order:

6 $${\partial }_{z}[{({h}_{f}^{(0)})}^{2}{u}_{f}^{\mathrm{(0)}}]=\mathrm{0,}$$

7 $${\partial }_{z}[{({h}_{f}^{\mathrm{(0)}}{u}_{f}^{\mathrm{(0)}})}^{2}]={\partial }_{z}(3{\rm{Oh}}{({h}_{f}^{\mathrm{(0)}})}^{2}{\partial }_{z}{u}_{f}^{\mathrm{(0)}}+{h}_{f}^{\mathrm{(0)}}[\frac{1+{({\partial }_{z}{h}_{f}^{\mathrm{(0)}})}^{2}+{h}_{f}^{\mathrm{(0)}}{\partial }_{zz}{h}_{f}^{\mathrm{(0)}}}{{\sqrt{1+{({\partial }_{z}{h}_{f}^{\mathrm{(0)}})}^{2}}}^{3}}])\mathrm{.}$$

The axial velocity *u*_*f*_^(0)^(*z*) can be solved in terms of *h*_*f*_^(0)^(*z*) from Eq. (6) using the boundary conditions, and then combined with Eq. (7) to obtain the second-order ordinary differential equation

8 $$\frac{1+6{\rm{Oh}}\cdot {h}_{f}^{\mathrm{(0)}}{\partial }_{z}{h}_{f}^{\mathrm{(0)}}}{{({h}_{f}^{\mathrm{(0)}})}^{3}}=\frac{1+{({\partial }_{z}{h}_{f}^{\mathrm{(0)}})}^{2}+{h}_{f}^{\mathrm{(0)}}{\partial }_{zz}{h}_{f}^{\mathrm{(0)}}}{{\sqrt{1+{({\partial }_{z}{h}_{f}^{\mathrm{(0)}})}^{2}}}^{3}}\mathrm{.}$$

**Figure 2:**
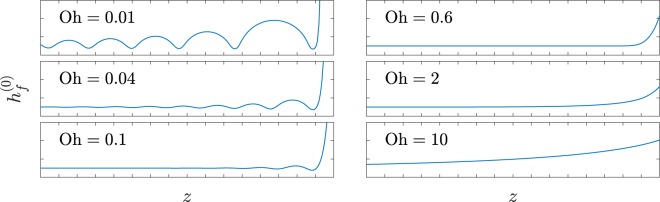
Numerical solutions for the profile of the steady filament region (via Eq. (8) with *z*_0_ = 0) for different Ohnesorge numbers. The distance between ticks is 2*R*. As Oh grows, solutions show slow decaying ripples (Oh = 0.01), fast decaying ripples (Oh = 0.1), fast smooth decay (Oh = 0.6) and slow smooth decay (Oh = 10.0). As explained in § 3.1, oscillations appear for ${\rm{Oh}} < {{\rm{Oh}}}^{\ast }={3}^{-\frac{1}{2}}$. At the *z* → ∞ limit, the solution shows the exponential behaviour of Eq. (9), with steeper growths for smaller Oh numbers.

This equation can only be integrated numerically, but several of its properties can be studied analytically. First, as expected, it is invariant under translations. Second, it can be shown that all solutions diverge for a finite *z* except for one, which is the one to be matched to a growing blob (see Supplementary Information). The analytic expansion of this solution on the right (around *z* → ∞) is

9 $$\mathop{\mathrm{lim}}\limits_{z\to \infty }{h}_{f}^{\mathrm{(0)}}(z)={{\rm{e}}}^{\frac{z-{z}_{0}}{\sqrt{3{\rm{Oh}}}}}+{\mathscr{O}}({{\rm{e}}}^{-\frac{z-{z}_{0}}{\sqrt{3{\rm{Oh}}}}}),$$

where *z*_0_ is a translational invariance constant. A set of these solutions, for different Ohnesorge numbers, is shown in Fig. 2. Depending on the Oh value, this region is either featured by decaying ripples (capillary waves) or a smooth spatial decay. These features are further analysed in Section §3.1.

### The Growing Blob

The blob region is characterised by two quantities: a time-dependent length scale, its radius *R*_*b*_(*t*), and the constant liquid mass influx as the filament recedes, *h*^2^*u* = 1. Consequently, the following self-similar solutions are proposed:

10 $${h}_{b}(z,t)={R}_{b}[{h}_{b}^{(0)}(\zeta )+{h}_{b}^{(1)}(\zeta ,t)+\ldots ],$$

11 $${u}_{b}(z,t)={R}_{b}^{-2}[{u}_{b}^{(0)}(\zeta )+{u}_{b}^{(1)}(\zeta ,t)+\ldots ],$$

where *ζ* ≡ *R*_*b*_^−1^*z*, and *h*_*b*_^(1)^(*ζ*, *t*) and *u*_*b*_^(1)^(*ζ*, *t*) are higher-order corrections in *t*. Combining these solutions with Eqs. (2) and (3), the following leading-order relationships are obtained:

12 $$2{R}_{b}^{2}{\dot{R}}_{b}{({h}_{b}^{\mathrm{(0)}})}^{2}-2{R}_{b}^{2}{\dot{R}}_{b}\zeta {h}_{b}^{\mathrm{(0)}}{\partial }_{\zeta }{h}_{b}^{\mathrm{(0)}}+{\partial }_{\zeta }[{({h}_{b}^{\mathrm{(0)}})}^{2}{u}_{b}^{\mathrm{(0)}}]=\mathrm{0,}$$

13 $${\partial }_{\zeta }(\frac{1+{({\partial }_{\zeta }{h}_{b}^{\mathrm{(0)}})}^{2}+{h}_{b}^{\mathrm{(0)}}{\partial }_{\zeta \zeta }{h}_{b}^{\mathrm{(0)}}}{{\sqrt{1+{({\partial }_{\zeta }{h}_{b}^{\mathrm{(0)}})}^{2}}}^{3}})=0,$$

where ${\dot{R}}_{b}\equiv {\partial }_{t}{R}_{b}$. Under this asymptotic scaling, Eq. (13) is uncoupled from Eq. (12) and the shape of the blob is given by the capillary term of the lubrication Eq. (3). The solution of Eq. (13) can be easily obtained, and is given by the semicircle

14 $${h}_{b}^{\mathrm{(0)}}(\zeta )=\sqrt{2\zeta -{\zeta }^{2}\mathrm{.}}$$

This is consistent with a uniform pressure inside the blob that slowly decays in time as the blob grows. The solution (14) has been chosen so that the left tip of the semicircle coincides with the origin. Replacing this solution in Eq. (12), and forcing the flux (*h*_*b*_^(0)^)^2^*u*_*b*_^(0)^ at the tip (*ζ* = 2) to be zero, we obtain a condition for the time-dependent length scale *R*_*b*_(*t*), i.e. ${R}_{b}^{2}{\dot{R}}_{b}=1/4$. Importantly, this condition is identical to the classical Taylor-Culick argument that balances the growth of the blob radius with the filament collected volume: ∂_*t*_(4*πR*_*b*_^3^/3) = *π*. Hence, in the asymptotic limit, the radius of the blob grows as *R*_*b*_(*t*) → (3*t*/4)^1/3^. The blob shape in terms of the variables *z* and *t* is given by

15 $${h}_{b}^{\mathrm{(0)}}(z,t)=\sqrt{2{R}_{b}(t)z-{z}^{2}},$$

where *h*_*b*_^(0)^(*z*, *t*) ≡ *R*_*b*_*h*_*b*_^(0)^(*ζ*). The position of the blob’s tip is given by *z*_*T*_ = 2*R*_*b*_(*t*), which properly satisfies the boundary conditions introduced in Section § 2. The expansion of Eq. (15) on the left is

16 $$\mathop{\mathrm{lim}}\limits_{z\to {0}^{+}}{h}_{b}^{\mathrm{(0)}}(z,t)={6}^{\mathrm{1/6}}{t}^{\mathrm{1/6}}{z}^{\mathrm{1/2}}+{\mathscr{O}}({t}^{-\mathrm{1/6}}{z}^{\mathrm{3/2}})\mathrm{.}$$

Another important observation is that the blob, in the asymptotic limit, is completely independent from viscosity, as evidenced by the momentum Eq. (13). Physically, the enlargement of the spatial scales in the growing blob slows the flow into a quasi-steady state that leads to the rise of surface tension as the unique dominant force.

### Intermediate matching zone

An intermediate region is required to match the zeroth-order solutions of the steady filament and the growing blob obtained in the previous sections. At the leading order, the profile of this intermediate zone has to fit both the steady-filament solution on the left ($h\propto \exp [z/\sqrt{3{\rm{Oh}}}]$) for *z* → −∞, and the blob on the right (*h* ∝ *t*^1/6^*z*^1/2^) for *z* → ∞. Provided that the flux *h*^2^*u* = 1 is conserved across the region, we propose the following ansatz:

17 $${h}_{m}(z,t)={t}^{\mathrm{1/6}}[{h}_{m}^{(0)}(\xi )+{h}_{m}^{(1)}(\xi ,t)+\ldots ],$$

18 $${u}_{m}(z,t)={t}^{-\mathrm{1/3}}[{u}_{m}^{(0)}(\xi )+{u}_{m}^{(1)}(\xi ,t)+\ldots ],$$

where *ξ* ≡ *z* is merely a spatial variable, and *h*_*m*_^(1)^(*ξ*, *t*) and *u*_*m*_^(1)^(*ξ*, *t*) are higher-order corrections. Combining these expressions with Eqs. (2) and (3), we obtain at the leading order:

19 $${\partial }_{\xi }[{({h}_{m}^{\mathrm{(0)}})}^{2}{u}_{m}^{\mathrm{(0)}}]=\mathrm{0,}$$

20 $${\partial }_{\xi }\,[{h}_{m}^{\mathrm{(0)}}(\frac{{\partial }_{\xi }{({h}_{m}^{\mathrm{(0)}})}^{2}+{h}_{m}^{\mathrm{(0)}}{\partial }_{\xi \xi }{h}_{m}^{\mathrm{(0)}}}{{({\partial }_{\xi }{h}_{m}^{\mathrm{(0)}})}^{3}})]+3{\rm{Oh}}{\partial }_{\xi }[{({h}_{m}^{\mathrm{(0)}})}^{2}{\partial }_{\xi }{u}_{m}^{\mathrm{(0)}}]=0.$$

Equation (19) can easily be integrated by taking the flux boundary condition ${({h}_{m}^{(0)})}^{2}{u}_{m}^{(0)}{|}_{\xi =-\infty }=1$. This yields *u*_*m*_^(0)^ = (*h*_*m*_^(0)^)^−2^, which can then be plugged into Eq. (20) to obtain the following equation

21 $$6{\rm{Oh}}{({\partial }_{\xi }{h}_{m}^{\mathrm{(0)}})}^{4}={({h}_{m}^{\mathrm{(0)}})}^{2}[{({\partial }_{\xi }{h}_{m}^{\mathrm{(0)}})}^{2}+{h}_{m}^{\mathrm{(0)}}{\partial }_{\xi \xi }{h}_{m}^{\mathrm{(0)}}]\mathrm{.}$$

This ordinary differential equation can be written as

$$6{\rm{Oh}}\frac{{\partial }_{\xi }{h}_{m}^{\mathrm{(0)}}}{{({h}_{m}^{\mathrm{(0)}})}^{5}}=\frac{{({\partial }_{\xi }{h}_{m}^{\mathrm{(0)}})}^{2}+{h}_{m}^{\mathrm{(0)}}{\partial }_{\xi \xi }{h}_{m}^{\mathrm{(0)}}}{{({h}_{m}^{\mathrm{(0)}}{\partial }_{\xi }{h}_{m}^{\mathrm{(0)}})}^{3}},$$

which can be integrated imposing a limiting condition matching the growing blob region, i.e. *h*_*m*_^(0)^ → *αξ*^1/2^ at *ξ* → ∞. This yields

$$\frac{3{\rm{Oh}}}{{({h}_{m}^{\mathrm{(0)}})}^{4}}=\frac{1}{{[{\partial }_{\xi }{h}_{m}^{\mathrm{(0)}}{h}_{m}^{\mathrm{(0)}}]}^{2}}-\frac{4}{{\alpha }^{4}},$$

where *α* is an integration constant. Using an auxiliary function *H*

22 $${h}_{m}^{\mathrm{(0)}}(\xi )=\alpha {(\frac{3}{4}{\rm{Oh}})}^{\mathrm{1/4}}H(\Xi \equiv \frac{2\xi }{\sqrt{3{\rm{Oh}}}}),$$

a first-order ordinary differential equation is obtained, this is

$$\frac{1}{{H}^{4}}=\frac{1}{4{(H{\partial }_{\Xi }H)}^{2}}-\mathrm{1,}$$

whose solution is given implicitly by the equation

$$\Xi =\sqrt{{H}^{4}+1}-\arctan (\frac{1}{\sqrt{{H}^{4}+1}}).$$

Expanding equation (22) into its two corresponding limits on the left and on the right, we respectively obtain

23 $$\mathop{\mathrm{lim}}\limits_{z\to -\infty }{h}_{m}^{\mathrm{(0)}}(z,t)=q{t}^{\mathrm{1/6}}{{\rm{e}}}^{\frac{z}{\sqrt{3{\rm{Oh}}}}}+{\mathscr{O}}({t}^{\mathrm{1/6}}{{\rm{e}}}^{\frac{5z}{\sqrt{3{\rm{Oh}}}}}),$$

24 $$\mathop{\mathrm{lim}}\limits_{z\to +\infty }{h}_{m}^{\mathrm{(0)}}(z,t)=\alpha {t}^{\mathrm{1/6}}{z}^{\mathrm{1/2}}+{\mathscr{O}}({t}^{\mathrm{1/6}}{z}^{-\mathrm{3/2}}),$$

where *h*_*m*_^(0)^(*z*, *t*) ≡ *t*^1/6^*h*_*m*_^(0)^(*ξ*) and *q* ≡ *α*(3e^−2^Oh)^1/4^. Equations (23) and (24) have already been written in terms of the original physical variables. It is important to point out that the intermediate matching region arises from the balance between viscous and capillary forces as shown by Eq. (20). Nonetheless, as the filament becomes thicker, the capillary force overcomes viscosity leading to a viscosity-independent behaviour, suitable to be matched with the growing blob. It should also be noted that the viscosity/surface tension balance in this region is the same used by Eggers & Fontelos^17^ to show that stretched very viscous drops never break up.

## The Evolving Shape of a Filament

**Figure 3:**
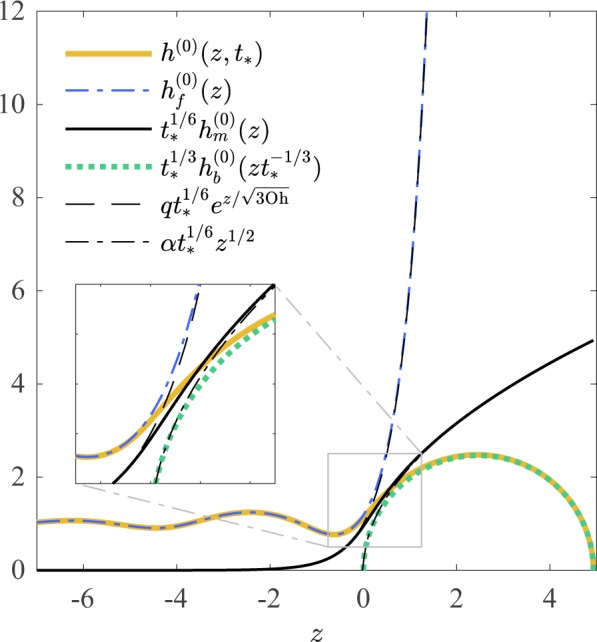
Asymptotic solutions for the asymptotic matching of the steady filament, the intermediate and circular blob regions for Oh = 0.1 and *t* = t_*_ = 20 in dimensionless units. The asymptotic tails beyond the matched regions are also shown.

**Figure 4:**
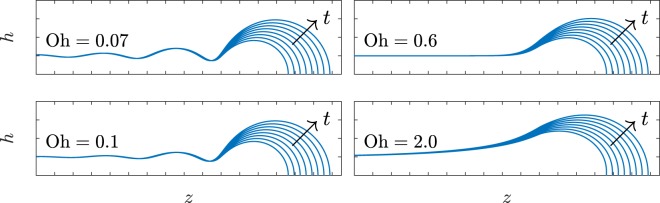
Filament profiles at *t* = 7.94, *t* = 10.25, *t* = 12.96, *t* = 16.12, *t* = 19.75, *t* = 23.88, *t* = 28.56 and *t* = 33.81 in dimensionless units for Oh = 0.07, Oh = 0.10, Oh = 0.60 and Oh = 2.00 in a Taylor-Culick velocity frame of reference. Curves were obtained following the asymptotic approach of Eq. (25). Here, the tick distance is equal to *R*.

The matching of the intermediate region solution, through Eqs. (23) and (24), with the far-field and blob solutions, via Eqs. (9) and (16), sets the coefficients *α* = 6^1/6^ and ${z}_{0}=-\sqrt{3{\rm{O}}{\rm{h}}}\mathrm{ln}(q{t}^{1/6})$. Notice that the slow dependence of *z*_0_ on time only modifies higher-order equations for *h*_*f*_^(1)^, leaving Eqs. (6) and (7) unaffected. Hence, the solution of leading order in time for the filament profile that satisfies the lubrication Eqs. (2) and (3) with the proper boundary conditions is

25 $${h}^{(0)}(z,t)={h}_{f}^{(0)}(z)+{t}^{\mathrm{1/6}}{h}_{m}^{(0)}(z)+{t}^{\mathrm{1/3}}{h}_{b}^{(0)}(z{t}^{-\mathrm{1/3}})-q{t}^{\mathrm{1/6}}{{\rm{e}}}^{\frac{z}{\sqrt{3{\rm{Oh}}}}}-\alpha {t}^{\mathrm{1/6}}{z}^{\mathrm{1/2}},$$

for *z* ≤ *z*_*T*_(*t*) = 2*R*_*b*_(*t*). This expression is valid under the assumption $t\gg 1$. A second criterion for the convergence of the solutions, $t\gg {({\rm{Oh}}\mathrm{/9.69})}^{3}$ can be obtained by analysing the following order (see Supplementary Information). An example of the leading order solution for a given time, including local solutions and asymptotic matched limits, is shown in Fig. 3. Evolving solutions based on Eq. (25) are depicted in Fig. 4. It is remarkable that the global solution of Eq. (25) has the expected behaviour even for values of *t* slightly above unity. Leading order expressions for *u*(*z*, *t*) can be obtained under the same framework, and the pressure inside the filament can be directly derived via the formalism of Eggers & Dupont^15^. The filament pressure *p* is shown as the colour scheme in Fig. 1. Additionally, higher-order corrections can be calculated too, following the scheme for the 2D case presented by Gordillo *et al*. in 2011^18^.

### Capillary waves and the decay length

**Figure 5:**
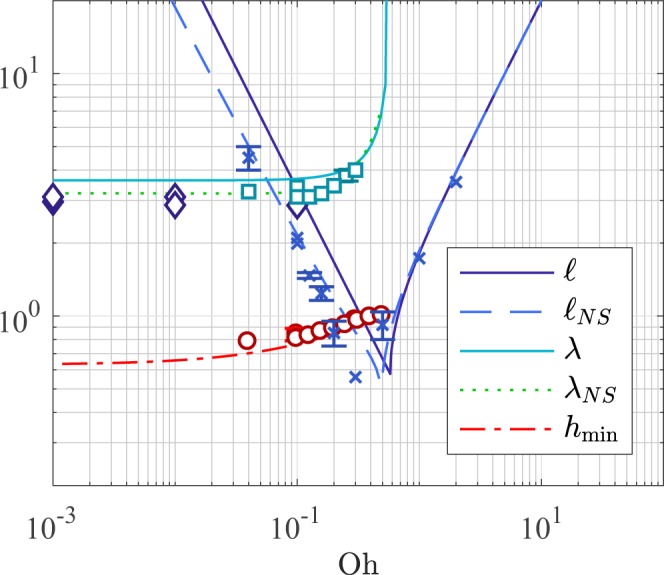
Wavelength, decay length, and neck thickness of the asymptotic solutions of lubrication equations as functions of Oh. Additionally, the wavelength and decay length obtained from linearisation of full Navier-Stokes equations are shown as dashed lines. Numerical results are shown for comparison, wavelengths from Notz & Basaran^6^ (◊) and Basilisk (□), decay lengths from Basilisk (×), and neck thickness from Basilisk (◦). Error bars are not shown when these are smaller than the symbol size.

The recoiling filament solution features travelling capillary waves that escape from the growing blob at the Taylor-Culick velocity. In the chosen frame of reference, the capillary waves are steady and hence can be approximated by ${h}_{f}^{(0)}(z)\approx 1+\varepsilon {e}^{-ikz}$, with $\varepsilon \ll 1$. Combining this with Eq. (8) and linearising, leads to the capillary wavenumber

26 $$k=3i{\rm{O}}{\rm{h}}\pm \sqrt{3-9{{\rm{O}}{\rm{h}}}^{2}},$$

whose values undergo a transition between complex and pure imaginary values at the threshold ${{\rm{Oh}}}^{\ast }={3}^{-\frac{1}{2}}\approx \mathrm{0.577.}$ For Oh ≥ Oh^*^, the wavenumber *k* is strictly imaginary and thus an exponential decay is observed as *z* → −∞. In contrast, for Oh < Oh^*^, *k* has a real component and the filament develops the well-known capillary waves modulated by the exponential decay. The dimensionless wavelength of these capillary ripples is given by *λ* = 2*π*/Re*(k*), which as Oh → 0, takes the value of ${\lambda }_{Oh\mathrm{=0}}=2\pi /\sqrt{3}\approx 3.63$. The linear analysis also reveals that the decay length, $\ell =\mathrm{1/}{\rm{Im}}(k)$, decays as (3Oh)^−1^ for low Ohnesorge numbers, and as (2Oh) in the large-Oh limit. Figure 5 shows the wavelength *λ* and decay length $\ell$ as functions of Oh.

The dispersion relation of the linearised Navier-Stokes equation for viscous filaments obtained by Rayleigh^19^ was used to validate our analytic results, which are based on the simplified lubrication model, Eqs. (2) and (3). In this analysis, we looked for waves whose phase velocity, *ω*/*k*, was equal to the Taylor-Culick velocity given in Eq. (1). Results are shown as dashed lines in Fig. 5. The qualitative agreement between the linearised lubrication approximation and full linearised Navier-Stokes equations is remarkably good, showing similar thresholds and asymptotic limits. Furthermore, the agreement is quantitatively excellent for Oh > 1.

### Neck thickness

A direct consequence of the existence of the capillary waves on the filament is the appearance of the *neck*, i.e. a finite global minimum right behind the blob. Figure 5 shows the filament neck thickness *h*_min_ as a function of the Ohnesorge number. The results show that as Oh decreases, *h*_min_ converges to a finite value. This value can be obtained by setting Oh = 0 in Eq. (8), and then integrating along *z* using the boundary condition *h* → ∞ as *z* → ∞. The result yields $\sqrt{1+{({\partial }_{z}h)}^{2}}=4{h}^{3}$. Setting ∂_*z*_*h* = 0, we obtain an equation for the minimum neck, whose solution is

27 $$\mathop{\mathrm{lim}}\limits_{{\rm{Oh}}\to 0}{h}_{min}={4}^{-\frac{1}{3}}\approx \mathrm{0.63,}$$

which correctly matches the curve trend shown in Fig. 5. It is important to note that the asymptotic solution in this report does not display any critical Ohnesorge value at which *h*_min_ → 0. This is contrary to what is observed in experiments and numerical simulations, in which sufficiently long filaments neck to the point of breakup during the retraction for ${\rm{Oh}}\,\lessapprox \,1$^20^. Indeed, this suggests that pinch-off might occur through a dynamic instability that precedes the asymptotic state.

## Validation

We analysed existing data in the literature for the purpose of comparisons with our findings. In the work of Notz & Basaran^6^, simulations of long liquid filaments were performed at Oh = 0.001, 0.010, 0.100, and 1.000. Capillary waves were seen for all the cases except for Oh = 1.000, implying that the critical limit is somewhere in the range of 0.1 < Oh^*^ < 1.0, which is well in agreement with our critical value of Oh^*^ = 0.577. Additionally, filament profiles from Notz & Basaran^6^ were analysed by image analysis showing capillary wavelengths in the region of *λ* = 2.8*R* to 3.1*R*. In fact, *λ* = (2.96 ± 0.15)*R* for Oh = 0.001, which is slightly lower than our inviscid value of *λ*_Oh=0_ = 3.63*R*. Further Ohnesorge values were explored by full 3D axisymmetric Navier-Stokes simulations using the flow solver Basilisk^21^. This solver uses adaptive mesh refinement and interface tracking with the volume of fluid (VoF) method. The initial configuration consisted of a square domain containing a semi-infinite free liquid cylinder of radius *R* surrounded by a passive gas (air). The simulations were executed in a comoving frame of reference receding at the Taylor-Culick speed. Dirichlet boundary conditions for the velocity were set on the left boundary, while outlet conditions were set on the other sides of the domain. All variables were non-dimensionalised with respect to the filament radius *R* and the liquid properties.

We ran simulations for filaments at several values of Oh to the point where the profile behind the blob remained steady (asymptotic regime). In our analysis, for Oh > 1.0, we estimated the decay length behind the blob using an exponential fitting. For Oh ≤ 0.5, we estimated the wavelength and decay length from the profile local maxima and minima behind the blob. These results are shown in Fig. 5, and show a good quantitative agreement with our model.

## Conclusions

In this report, we have derived a first-order asymptotic expansion for a Newtonian liquid filament. Our analysis identifies three distinct regions: a steady filament section, an expanding spherical blob, and an intermediate *matching* zone. Importantly, we have shown that, below a critical Ohnesorge number, capillary waves naturally emerge along the surface of the filament. We have analysed both their wavelength and spatial decay length along the filament as functions of Oh. We analytically prove that, at the inviscid limit, the wavelength of the capillary waves is 3.63 times the filament’s radius. Our findings are found to be in agreement with numerical results in Basilisk and those corresponding to the seminal work of Notz & Basaran^6^.

## Supplementary information

Supplementary Material
