# Supplementary material for: Shape of a recoiling liquid filament
Source: Sci Rep. 2019 Oct 29;9:15488. doi: 10.1038/s41598-019-51824-3 (PMC6820550; doi:10.1038/s41598-019-51824-3)
Supplement: Supplementary file 1 — Supplementary Material [file 41598_2019_51824_MOESM1_ESM.pdf]

Supplementary Material
"Shape of a recoiling liquid filament"

Francesco Paolo Contò[1], Juan F. Marín[2], Arnaud Antkowiak[3], J. Rafael Castrejón-Pita[1], and Leonardo Gordillo[2,*]

[1]School of Engineering and Materials Science, Queen Mary University of London, London E1 4NS, United Kingdom.
[2]Departamento de Física, Universidad de Santiago de Chile, Av. Ecuador 3493, Estación Central, Santiago, Chile.
[3]Institut Jean Le Rond d'Alembert, UMR 7190 CNRS/UPMC, Sorbonne Universités, F-75005 Paris, France.
[*]leonardo.gordillo@usach.cl

Separatrix of steady solutions

In brief, here we show that a single solution of equation (8) exists that does not diverge for finite z. Figure S1 presents numerical solutions of equation (8) for different initial conditions, i.e. $h_f^{(0)}(z_0)$ and $\partial_z h_f^{(0)}|_{z=z_0}$, at $z_0 = 0$. This way, Figure S1(a) shows the phase diagram $\partial_z h_f^{(0)}$ in terms of $h_f^{(0)}(z)$, at $\mathrm{Oh} = 0.4$. Here, two different families of solutions can be easily identified; both of them being singular, i.e. $\partial_z h_f^{(0)}$ diverges for finite values of $h_f^{(0)}$. The first family, plotted in green lines, corresponds to profiles with a diverging positive slope. In contrast, the second family, plotted as blue lines, corresponds to profiles with a diverging negative slope. Figure S1(b) shows the behaviour of these solutions in the physical space ($h_f^{(0)}$ as a function of z), at $\mathrm{Oh} = 0.1$. The points of divergence are marked with a symbol ($\times$) and, as seen, occur at a finite z. Similar behaviour is observed at any Ohnesorge number.

The solutions, illustrated in Figure S1, reveal that a *separatrix* (a boundary separating the two behaviours) divides both families; the separatrix is plotted as a thick black line. The asymptotic expansion of the solution around $z \to +\infty$ can be found by setting $h_f^{(0)}, \partial_z h_f^{(0)}, \partial_{zz} h_f^{(0)} \gg 1$ in equation (8), yielding

$$\frac{6\mathrm{Oh} \cdot h_f^{(0)} \partial_z h_f^{(0)}}{\left[h_f^{(0)}\right]^3} = \frac{\left[\partial_z h_f^{(0)}\right]^2 + h_f^{(0)} \partial_{zz} h_f^{(0)}}{\left[\partial_z h_f^{(0)}\right]^3}.$$

This solution provides the leading term of equation (9); higher-order terms are found as perturbations from equation (8).

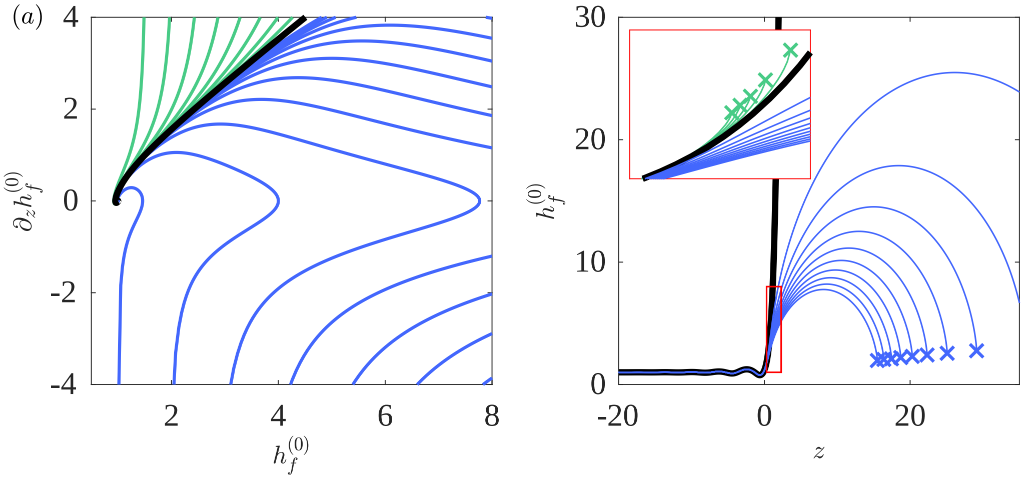

Supplementary Figure S1. (a) Phase diagram of the singular solutions of Eq. (8) obtained through numerical integration at different initial conditions, here $\mathrm{Oh} = 0.4$. Two family solutions are found (green and blue) corresponding to singularities with different diverging slopes: the green curves present positive divergence and the blue curves show negative divergence. A *separatrix*, seen as a thick black curve, is the unique solution whose derivative only diverges when $h_f^{(0)} \to +\infty$. (b) Spatial profiles at $\mathrm{Oh} = 0.1$.

Additional criterion for the validity of the asymptotic solutions

A second criterion arises from the higher-order corrective terms of the three regions (equations 9, 16 and 24) and shows that the largest higher-order term is that of the intermediate zone, i.e. $\mathscr{O}\left(t^{1/6}z^{-3/2}\right)$ of equation 24. This term, calculated from expanding equation (22), is given by

$$\frac{3 \cdot \mathrm{Oh}}{16}\alpha t^{1/6} z^{-3/2}.$$

In the blob, $z \sim t^{1/3}$, so the decay of this correction as time evolves is guaranteed. Furthermore, one can evaluate the small residue that arises at the right tip of the filament, $z = 2R_b(t) = \alpha^2 t^{1/3}$, which is equal to

$$\frac{3 \cdot \mathrm{Oh}}{16\alpha^2} t^{-1/3},$$

which should be much smaller than 1 for a good approximation. Consequently, the criterion for the convergence of the solutions, provided that $\alpha = 6^{1/6}$, is

$$t \gg t_\infty \equiv \left(\frac{9}{8192}\right)\mathrm{Oh}^3 \approx \left(\frac{\mathrm{Oh}}{9.69}\right)^3,$$

which is a function of the Ohnesorge number. The shortest time used in the paper is $t = 7.94$ (at $\mathrm{Oh} = 2$): according to our criterion, this leads to $t_\infty = 0.009$ which accordingly fulfills the condition. Noteworthy, this criterion becomes dominant over $t \gg 1$ for $\mathrm{Oh} \gtrapprox 9.69$, and is equivalent to the condition

$$R_b(t) \gg \frac{\mathrm{Oh}}{10.67},$$

which follows from $R_b \sim (3t/4)^{1/3}$.
